# Supplementary material for: Causal Relationship between Aging and Anorexia Nervosa: A White-Matter-Microstructure-Mediated Mendelian Randomization Analysis
Source: Biomedicines. 2024 Aug 16;12(8):1874. doi: 10.3390/biomedicines12081874 (PMC11351342; doi:10.3390/biomedicines12081874)
Supplement: Supplementary file 1 [file biomedicines-12-01874-s001.zip › Supplementary Figure/Figure S8/Leaveoneout plot of MR analysis for TL to ubm-b-1994.pdf]

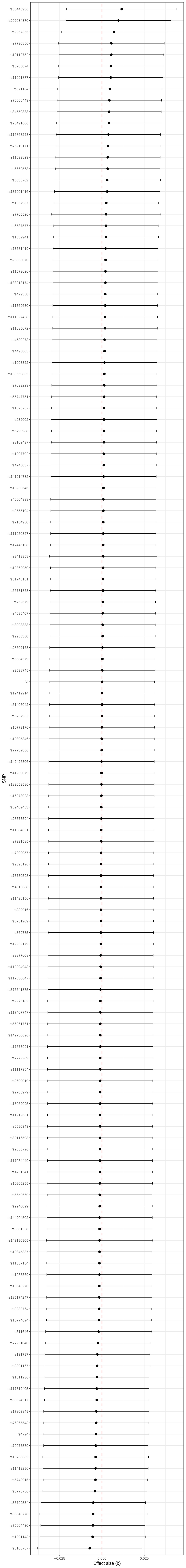

rs35446936
rs202034370
rs2967355
rs7790856
rs10112752
rs3785074
rs11991877
rs871134
rs76666449
rs34550383
rs78491606
rs116863223
rs76219171
rs11699829
rs6669563
rs6536702
rs137901416
rs1957937
rs7705526
rs6587577
rs1332941
rs73581419
rs28363070
rs11579626
rs188918174
rs429358
rs11769630
rs111527438
rs11085072
rs4530278
rs4498805
rs1003322
rs139669835
rs7099229
rs55747751
rs1023767
rs932002
rs6790988
rs8102497
rs1907702
rs4743037
rs141214782
rs13230646
rs45604339
rs2555104
rs7164950
rs111950327
rs17445108
rs9419958
rs12369950
rs61748181
rs66731853
rs782679
rs4695407
rs3093888
rs9955360
rs28502153
rs6584579
rs2538745
All
rs12412214
rs61405042
rs3767952
rs10773176
rs10805346
rs77732866
rs142426306
rs41269079
rs182059586
rs16978028
rs59409453
rs28577594
rs11584821
rs7221585
rs7209057
rs9398196
rs73730598
rs4616688
rs11426156
rs939916
rs6751209
rs869785
rs12932179
rs2977606
rs112394943
rs117630647
rs376641875
rs2276182
rs117407747
rs56061761
rs142730696
rs17677991
rs7772289
rs11117354
rs9600019
rs2763979
rs13062095
rs11212631
rs6590343
rs80116508
rs2056726
rs117034449
rs4731541
rs10905255
rs6659669
rs9940099
rs144204502
rs6881568
rs143190905
rs10845387
rs11557154
rs1985369
rs10840270
rs185174247
rs2282764
rs10774624
rs611646
rs77231040
rs131797
rs3891167
rs1611236
rs117512405
rs80324517
rs17803849
rs76065543
rs4724
rs79977579
rs10768683
rs11412296
rs5742915
rs6776758
rs56799554
rs35640778
rs75664430
rs1291143
rs8105767
SNP
-0.025
0.000
0.025
Effect size (b)
